# Bradycardias in Patients with Pulmonary Hypertension—Prevalence, Pathophysiology and Clinical Relevance

**DOI:** 10.3390/jcdd12040120

**Published:** 2025-03-28

**Authors:** Paul Ole Behrendt, Lukas Ley, Hossein Ardeschir Ghofrani, Dirk Bandorski

**Affiliations:** 1 Faculty of Medicine, Semmelweis University Campus Hamburg, 20099 Hamburg, Germany; paul.behrendt@semmelweis-hamburg.de; 2 Campus Kerckhoff, Justus-Liebig-University Giessen, 61231 Bad Nauheim, Germany; lukas.m.ley@med.uni-giessen.de; 3 Department of Internal Medicine, Justus-Liebig-University Giessen, Universities of Giessen and Marburg Lung Center (UGMLC), 35392 Giessen, Germany; ardeschir.ghofrani@innere.med.uni-giessen.de; 4 Kerckhoff Heart and Thorax Center, Department of Pneumology, 61231 Bad Nauheim, Germany; 5 Department of Medicine, Imperial College London, London SW7 2AZ, UK

**Keywords:** pulmonary hypertension, PH, arrhythmia, bradycardia, bradyarrhythmia, bradycardic arrhythmia

## Abstract

Introduction: Arrhythmias are a frequent complication of pulmonary hypertension (PH). Supraventricular tachycardias (SVT) are predominantly reported and are associated with clinical deterioration and an increased mortality. In contrast, the prevalence and clinical relevance of bradycardias is largely unclear. Therefore, the aim of the present study was to determine a prevalence of bradycardias in PH patients and to outline their clinical relevance. Material and methods: Between January 2000 and June 2013, consecutive PH patients were pro- and retrospectively enrolled in two cohorts. Patients received either a 24 h or 72 h Holter ECG. Results: A total of 314 patients (58% female, mean age: 63 years) from PH groups 1–5 (39%, 11%, 19%, 28%, 3%) were included. Basic heart rhythm was sinus rhythm in 87% of patients (9% atrial fibrillation, 2% atrial flutter and 2% paced rhythm). Further arrhythmias were detected in 34% of patients (SVT: 12%, non-sustained ventricular tachycardia: 16%) with a 6% prevalence of relevant bradycardias. Atrioventricular block was revealed in 5% of patients (seven first-degree, one and three second-degree Wenckebach and Mobitz type, respectively, four third-degree), and 1% revealed sinoatrial block (one second-degree, third-degree and unspecified each). Conclusions: The prevalence of bradycardias appears to be about 5–10% in PH patients. Most of them are short and self-limiting. However, some patients experience syncope or clinical deterioration and, therefore, need specific treatment. To find these patients, long-term ECG monitoring combined with ECG-symptom correlation may be useful. Bradycardic medication should be excluded as a cause.

## 1. Introduction

Pulmonary hypertension (PH) is a cardiopulmonary disease characterized by an increase in mean pulmonary artery pressure (mPAP) of >20 mmHg at rest, assessed by right heart catheterization (RHC). Based on the underlying pathophysiology, PH is classified into five groups [1]. Their commonality is an increase in right ventricular afterload, which leads to right heart failure (RHF), consecutive distressing symptoms, a reduction in quality of life and a relevant mortality rate [1,2,3,4,5,6]. Arrhythmias are a significant complication mostly of advanced PH. Supraventricular tachycardias (SVT, prevalence: 8–46%), especially atrial fibrillation (AFib) and atrial flutter (AFlu), and non-sustained ventricular tachycardias (nsVTs, prevalence: 13–32%) are predominantly reported [1,7,8,9,10,11,12,13,14,15,16,17,18,19,20,21,22]. In PH patients, SVTs are associated with clinical deterioration, a prolonged hospitalization and, if persistent, with an increase in mortality [7,10,11,12,13,14,15,16,18,23]. However, restoration of the sinus rhythm improves the patient’s symptoms and prognosis significantly [7,10,11,14,18]. The prevalence and clinical relevance of bradycardias in PH, on the other hand, is largely unclear, and study results are scarce [24,25,26,27]. Therefore, the aim of the present study was to determine a prevalence of bradycardias in PH patients by analysis of own unpublished data, to compare it with the current literature and to discuss the clinical relevance of bradycardias in PH patients.

## 2. Materials and Methods

In the following sections, two different studies are described which have already been carried out in the past, but have never been analyzed nor published regarding the prevalence and clinical relevance of bradycardias in PH patients.

### 2.1. Study Design

#### 2.1.1. First Cohort

The first study was conducted as a unicenter, prospective study in a German high-volume PH referral center. Between July 2012 and February 2013, 362 consecutive PH patients attending PH outpatient clinic were analyzed regarding the inclusion criteria (PH diagnosis according to the 2009 ESC/ERS guidelines, ≥18 years of age, no known pre-existing arrhythmias and available written informed consent to participate in the study) and were included if they met all these criteria [28]. Subsequently, all patients underwent a 72 h Holter electrocardiogram (ECG). The aim of the study adapted for this analysis was to determine the prevalence and clinical relevance of arrhythmias, especially bradycardias, in PH patients. The study was conducted according to the guidelines of the Declaration of Helsinki and approved by the Institutional Review Board of the Justus-Liebig-University Giessen (#32/12, dated 24 April 2012).

#### 2.1.2. Second Cohort

This study was conducted as a unicenter, retrospective study in a German high-volume PH referral center. Between January 2000 and June 2013, 283 consecutive PH patients were analyzed regarding the inclusion criteria (PH diagnosis according to the 2009 ESC/ERS guidelines, 24 h Holter ECG performed, ≥18 years of age and available written informed consent to participate in the study) and were included if they met all criteria [28]. The aim of the study adapted for this analysis was to determine the prevalence and clinical relevance of arrhythmias, especially bradycardias, in PH patients. The study was conducted according to the guidelines of the Declaration of Helsinki and approved by the Institutional Review Board of the Justus-Liebig-University Giessen (#107/13, dated 19 August 2013).

### 2.2. Holter ECG Monitoring

For assessment of arrhythmias, all patients underwent a 24 h (cohort two) or 72 h (cohort one) Holter ECG (CardioMem^®^CM 3000, getemed, Teltow, Germany) during their daily routine. The recordings were analyzed by a board-certified cardiologist using an operator-controlled analysis (CardioDay^®^, getemed, Teltow, Germany). Holter ECGs were analyzed with standard nomenclature and definitions.

### 2.3. Statistical Analysis

A statistical analysis was performed with SPSS Statistics (Version 29, IBM, Armonk, NY, USA). Nominal variables are presented as numbers and percentages. The distribution was tested for normality. Continuous variables are presented as mean or median and standard deviation or interquartile range, as appropriate. Comparisons between groups were carried out using *t*-test or chi-square test as appropriate. A *p*-value of <0.05 was considered statistically significant.

## 3. Results

### 3.1. First Cohort

A total of 92 patients (72% female, mean age: 61 years) with PH groups 1, 3, 4 and 5 (59%, 11%, 28% and 2%) and without previously documented arrhythmias were included and underwent a 72 h Holter ECG. The average recording duration was 3849 min, i.e., 64 h and 9 min. Due to patient-related factors (premature detachment of the ECG electrodes or premature device removal by the patient), the recording duration was less than 72 h in 29% of the patients. Furthermore, at the time of recording 16% of patients received antiarrhythmic drug treatment with ß-blockers (14%) or digitalis (2%). During recording, 99% of patients were in sinus rhythm (1% AFlu). Eighteen percent of patients showed relevant arrhythmias. Three percent of patients had an intermittent third-degree atrioventricular (AV) block during the day with pauses from 2.2 to 5.5 s, which disappeared in one patient after discontinuation of the ß-blocker but was treated with pacemaker implantation in the other two patients. These two patients had not received any antiarrhythmic drugs or bradycardic medication. One percent of the patients showed a second-degree AV block Mobitz type. However, this patient refused pacemaker implantation. Patients with bradycardias compared to those without did not show any significant difference in clinical data (Table 1) apart from systolic pulmonary artery pressure (sPAP) and presence of chronic thromboembolic pulmonary hypertension (CTEPH). sPAP was statistically significantly lower in patients experiencing bradycardias (mean: 33 vs. 64 mmHg, *p* = 0.020), and patients with bradycardias more often suffered from CTEPH (75 vs. 26%, *p* = 0.034). Furthermore, 13% of patients showed nsVTs, and 1% of patients suffered from newly diagnosed AFlu.

### 3.2. Second Cohort

A total of 222 patients (53% female, mean age: 64 years) from PH groups 1–5 (31%, 16%, 22%, 28% and 2%) were included and underwent a 24 h Holter ECG. At the time of recording, patients received antiarrhythmic drug treatment with ß-blockers (25%), verapamil/diltiazem (19%), digitalis (19%), amiodarone (3%), flecainide (0.5%), ivabradine (0.5%) and/or propafenone (0.5%)—often in combination. During recording, sinus rhythm was the basic heart rhythm in 82% of patients (13% continuous AFib, 2% continuous AFlu, 3% continuously paced rhythm). In addition to pathological basic heart rhythms, relevant arrhythmias were detected in 41% of patients. AV blocks occurred in 5% of patients. Of the PH patients, 3% suffered from first-degree AV block, 1.5% from second-degree AV block Wenckebach (0.5%) and Mobitz type (1%), and 0.5% from third-degree AV block. Moreover, sinoatrial (SA) blocks were noted in 1.5%, including one second-degree, one third-degree SA block and one unspecified SA block each. Patients with bradycardias compared to those without did not show any significant difference in clinical data (Table 2) apart from sPAP and prescription frequency of verapamil/diltiazem. sPAP was statistically significantly lower in patients experiencing bradycardias (mean: 46 vs. 66 mmHg, *p* = 0.024) and verapamil/diltiazem was more frequently prescribed in patients with bradycardias (43 vs. 17%, *p* = 0.018). Moreover, SVT and nsVTs were detected in 17.5% and 17% of PH patients.

## 4. Discussion

As mentioned above, the data on the prevalence, clinical relevance and pathophysiology of bradycardias in PH patients are scarce. The two present studies showed a combined prevalence of relevant bradycardias of 6% in group 1–5 PH patients. In total, 5% of patients revealed AV block (seven first-degree, one and three second-degree Wenckebach and Mobitz type, respectively, four third-degree) and 1% revealed SA block (one second-degree, third-degree and unspecified each). Further literature confirms our data. The observed significant differences in echocardiographic sPAP between patients with or without bradycardias in both cohorts are most likely due to the small sample size of bradycardic patients (*n* = 18) and are most likely not clinically relevant. Although the total number of bradycardic patients is probably not high enough to draw strong and valid conclusions, the present data do show some remarkable features. Although the clinical data in bradycardic and non-bradycardic patients are largely the same in both cohorts, none of the bradycardic patients was a group 2 PH patient (PH associated with left heart disease) or a patient with a history of a congenital heart disease (pulmonary arterial hypertension associated with congenital heart disease [PAH-CHD], group 1). In addition, bradycardia (AV block III°) was indeed triggered by medication (ß-blockers) in one patient in cohort one and verapamil/diltiazem were prescribed significantly more frequently in bradycardic patients in cohort two (43 vs. 17%, *p* = 0.018). However, the overall prevalence of antiarrhythmic drugs was similar in bradycardic and non-bradycardic patients in both cohorts.

### 4.1. Current Study Situation on Bradycardias in PH Patients

In 1979, Kanemoto et al. reviewed twelve-lead ECGs from 101 pulmonary arterial hypertension (PAH) patients (73% female, mean age: 31 years) who were undergoing treatment. In 27% of patients, 34 different arrhythmias (34%) were found. Most frequently, sinus tachycardias (13%), sinus bradycardias (6%, heart rate: 45–58/min) and AV blocks (6% in total, 5% first-degree AV block and 1% second-degree AV block Wenckebach type) were found. However, all AV blocks were induced by digitalis for treatment of RHF. The authors did not note any more pronounced sinus bradycardias, SA blocks or sinus arrests. However, Kanemoto et al. did not state the specific symptoms of the patients suffering from theses arrythmias. Furthermore, they did not state whether the bradycardias required treatment and, if so, how they were treated. They only reported that the second-degree AV block disappeared after reduction of digitalis. Moreover, only ECGs of short duration were recorded (5–10 s per lead). Even the authors emphasized the following: “However, it is not clear whether or not the ECGs were recorded during a syncopal attack or chest pains and the method of detection of arrhythmias must be taken into consideration. […] Therefore, in the future investigation of such patients, it would be necessary to record ECGs using long-term monitoring” [24].

In another study, Bossone et al. reviewed twelve-lead ECGs from 51 newly diagnosed PAH patients (88% female, mean age: 42 years). They noted sinus bradycardia in 4% of patients, a first-degree AV block in 12% of patients, a junctional rhythm and an ectopic atrial rhythm in 2% each. Moreover, 2% of patients already had a dual-chamber pacemaker implanted. However, Bossone et al. did not state the specific symptoms of the patients suffering from theses arrhythmias. Furthermore, they also did not state whether the arrhythmias required treatment and, if so, how they were treated. They also did not mention the reason for pacemaker implantation. Moreover, only standard twelve-lead ECGs of short duration were recorded [25].

Interesting findings were made in a study by Andersen et al. They conducted a study on 34 PH patients (71% PAH and 29% CTEPH, 47% female, median age: 58 years) without previous arrhythmias diagnosed. All patients received an insertable cardiac monitor (ICM). The aim was “to provide a more detailed and contemporary description of arrhythmias in PH”. Total monitoring time was 46 patient-years (median: 594 days per patient). They captured 70 arrhythmic episodes in 38% of the patients. SVTs were the most common arrhythmias (35%) and accounted for >99% of arrhythmic time burden. The median time of arrhythmic burden was 1.6 min per patient. Only 9% of patients (3/34) experienced bradycardias (heart rate ≤30/min for at least four consecutive heart beats). In total, they detected five bradycardic episodes. In two patients, bradycardic episodes persisted for a duration of four seconds and manifested during nocturnal hours. In another patient, two episodes were recorded, resulting in syncope. One episode was characterized by an SA block that occurred during physical activity and persisted for a duration of 10 s. The other episode was characterized by an ectopic ventricular rhythm that persisted for a duration of 50 s. Accordingly, in 3% of patients, bradycardia resulted in a syncope and clinical deterioration necessitating the intensification of PAH therapy and the implantation of a dual-chamber pacemaker due to SA block. The absolute advantage of this study was the long-term (in median over one year per patient) continuous monitoring via ICM [26].

Based on the findings of the present cohorts and the current literature, the prevalence of bradycardias appears to be about 5–10% in PH patients (Table 3) [24,25,26]. A too-short recording period (12-lead ECG) could lead to an underestimation of their prevalence [24]. Nevertheless, Andersen et al. stated a prevalence of 9% when observing PH patients for a median of 594 days per patient by ICM [26]. However, since arrhythmic episodes are often short and self-limiting, the proportion of arrythmias necessitating treatment (e.g., modification of medication or pacemaker implantation) is probably considerably smaller [26]. An ECG-symptom correlation should be aimed for when evaluating therapeutic relevance. It should also be noted that PH patients often take bradycardic medication, which could partly explain the bradycardias. For example, in the present study one bradycardia (AV block III°) was indeed triggered by medication (ß-blockers) and was no longer detectable after ß-blocker discontinuation. Therefore, if distinct symptoms are present, the patient’s medication should be evaluated, and a long-term ECG monitoring (Holter ECG, ICM, wearables) should be considered to only treat clinically relevant bradycardias. Moreover, it could be assumed that other pre-existing heart diseases increase the risk of bradycardias in PH patients. However, the present study could not confirm this. No bradycardic patient suffered from group 2 PH or CHD-PAH.

### 4.2. Pathophysiology of Bradycardias in PH Patients

Hypotheses on the pathophysiology of SVTs in PH patients have been described in detail [29,30,31,32]. In contrast, there are only a few studies and hypotheses on the pathophysiology of bradycardias in PH patients.

In 2012, Medi et al. performed electrophysiologic studies on 8 PAH patients and 16 age-matched controls. They found out that PAH was associated with an electrical remodeling of the right atrium. Compared to the controls, PAH patients had, among other things, prolonged corrected sinus node recovery times (*p* = 0.02), lower conduction velocities (*p* < 0.001) and increased low voltage areas (*p* < 0.01) in the right atrium [33]. These findings are of particular relevance, because sinus node dysfunction (SND), potentially leading to bradycardias, was found to be associated with prolonged corrected sinus node recovery times, conduction delay along the cardiac conduction system and increased low voltage areas within the right atrium [34,35].

Moreover, Logantha et al. conducted a monocrotaline-rat model on the effects of PAH on sinus node function. PAH induction by injection of monocrotaline led to a remodeling of several genes in the sinus node. In detail, they observed that monocrotaline injection led to significant downregulation of many ion channels (responsible for the pacemaking process) and calcium-handling genes (responsible for the electromechanical coupling process) as well as a significant upregulation of many fibrosis genes in the sinus node of the rats, which could explain SND [36].

Temple et al. also conducted a monocrotaline-rat model to test the hypothesis that an AV block may be a result of a change in the ion channel transcriptome of the AV node. After causing PAH, AV node dysfunction was revealed alongside with a widespread downregulation of ion channels and related genes in the AV node which would result in AV block. In detail, the downregulation of specific L-type Ca^2+^ channels appeared to lead to a slowing or even blockade of AV conduction. Their conclusion was that “Pulmonary hypertension results in a derangement of the ion channel transcriptome in the AV node, and this is the likely cause of AV node dysfunction in this disease” [37].

Lastly, Hoeper et al. retrospectively evaluated clinical data of 3310 patients on the outcome of cardiopulmonary resuscitation (CPR) in patients with PAH. A total of 513 patients had circulatory arrest, and CPR was initiated in 132. They found out that 45% of the patients in whom CPR was initiated exhibited bradycardia (type of bradycardia not specified), and 15% showed asystole as the initial rhythm at the time of CPR. Furthermore, Hoeper et al. found out that CPR efforts were unsuccessful in 79% of patients, although 94% of the patients were hospitalized (74% in intensive care units or equally equipped facilities), and there was only minimal delay until CPR was started [27]. It could, therefore, be assumed that these bradycardias were a sign of a “dying heart” after suffering severe right heart strain for a long time.

All in all, PH appears to lead to an electrical remodeling of the right atrium as well as a remodeling of ion channel, calcium-handling and fibrosis genes in the SA and AV node which all might cause SND and AV block. However, it must be noted, that most of these observations were made in PAH animal models. A transferability to all PH groups in humans might not be possible. The exact pathophysiology of bradycardias in PH, therefore, remains unclear. However, as mentioned before, it should be noted that the pathophysiology of PH may not always be the only explanation for bradycardias in these patients. Since PH patients also frequently take negative chronotropic drugs (e.g., ß-blockers, verapamil/diltiazem or digitalis), this could also be a cause of bradycardias in PH patients and should be considered.

### 4.3. Limitations

The present study stands out due to several of its features (a large total PH sample size (*n* = 314), patients of all five PH groups, use of 24 and 72 h Holter ECGs, partly prospective study design) which potentially increase transferability and reduce confounders. Nevertheless, the present study also has some limitations, all of which could decrease its external validity and complicate transferability to the general population of PH patients. Conducting the studies in a single PH referral center with highly selected patients in a partly retrospective design could have led to potential biases (e.g., selection bias with hemodynamically more severely affected patients or over-representation of PH group 1 and PH group 4 patients). Moreover, some clinical data, such as symptoms (both cohorts), symptom-ECG correlation (both cohorts), indication for and exact number of patients receiving antiarrhythmic drugs (both cohorts) as well as treatment of bradycardias (cohort 2), were not available. Moreover, due to missing data the possible (side) effects of other drugs could not be evaluated, e.g., the use of diuretics might have caused electrolyte changes which could have contributed to the bradycardias. Furthermore, most likely because pre-existing arrhythmias were no exclusion criterion in cohort two, the frequency of negative chronotropic drug prescription was higher in cohort two. This might have led to a higher rate of bradycardias. However, our data blends in well with the data of other studies. In addition, due to the low prevalence of bradycardias, the total sample size may not be enough to provide sufficient data. In addition, the use of Holter ECGs is a strength compared to 12-lead ECGs. However, for an even more accurate determination of the prevalence of bradycardias, it would be even better to monitor the heart rhythm of PH patients over an even longer time span. ICM systems and the widely available one-channel ECG wearables could be used for this purpose in future studies.

## 5. Conclusions

Based on the findings of the present cohorts and the limited current literature, the prevalence of bradycardias appears to be about 5–10% in PH patients. Animal models suggest that an electrical remodeling of the right atrium as well as a remodeling of ion channel, calcium-handling and fibrosis genes in the SA and AV node might cause SND and AV blocks and, therefore, bradycardias in PH. However, the exact pathophysiology of bradycardias in PH remains unclear. Moreover, most of the bradycardias seem to be short and self-limiting. Nevertheless, some of the patients experience syncope or clinical deterioration and, therefore, need specific treatment (e.g., discontinuation of negative chronotropic agents, modification of PH medication or pacemaker implantation). To find these patients, long-term ECG monitoring (Holter ECG, ICM, wearables) combined with ECG-symptom correlation may be useful. In addition, bradycardic medication should be excluded as a cause. Future large, multicenter, prospective studies should comprise long-term continuous monitoring of the heart rhythm and aim to further clarify the exact prevalence and clinical significance of bradycardias (short and self-limiting vs. necessitating treatment) in PH patients.

## Figures and Tables

**Table 1:** Comparison of clinical data of patients with and without bradycardias in cohort one.

		Patients with Bradycardias	Patients Without Bradycardias	*p*-Value
Patients, *n*		4 (4)	88 (96)	-
Age, mean (SD)		60 (12)	61 (14)	0.865
Sex, female, *n* (%)		2 (50)	64 (73)	0.975
PH group, *n* (%)	1: IPAH	0 (0)	33 (38)	-
	1: CTD-PAH	1 (25)	9 (11)	0.862
	1: HIV-PAH	0 (0)	2 (2)	-
	1: PoH-PAH	0 (0)	4 (5)	-
	1: CHD-PAH	0 (0)	5 (6)	-
	2	0 (0)	0 (0)	-
	3	0 (0)	10 (11)	-
	4	3 (75)	23 (26)	0.034
	5	0 (0)	2 (2)	-
6MWD, m, mean (SD)		405 (138)	332 (159)	0.375
BNP, pg/mL, mean (SD)		140 (205)	110 (138)	0.680
sPAP ^1^, mmHg, mean (SD)		33 (6)	64 (26)	0.020
TAPSE, mm, mean (SD)		22 (1)	21 (5)	0.664
PVR, dyn × ec × cm^−5^, mean (SD)		404 (89)	572 (323)	0.470
Antiarrhythmic drugs, *n* (%)	ß-blocker	1 (25)	12 (14)	0.523
	digitalis	0 (0)	2 (2)	-
AV block, *n* (%)	I	0 (0)	0 (0)	-
	IIa	0 (0)	0 (0)	-
	IIb	1 (25)	0 (0)	-
	III	3 (75)	0 (0)	-
SA block, *n* (%)	I	0 (0)	0 (0)	-
	II	0 (0)	0 (0)	-
	III	0 (0)	0 (0)	-

Legend: AV: atrioventricular, BNP: brain natriuretic peptide, CHD-PAH: PAH associated with congenital heart disease, CTD-PAH: PAH associated with connective tissue disease, HIV-PAH: PAH associated with an human immunodeficiency virus infection, IIa: AV block Mobitz type, IIb: AV block Wenckebach type, IPAH: idiopathic PAH, PAH: pulmonary arterial hypertension, PH: pulmonary hypertension, PoH-PAH: PAH associated with portal hypertension, PVR: pulmonary vascular resistance, SA: sinoatrial, SD: standard deviation, sPAP: systolic pulmonary artery pressure, TAPSE: tricuspid annular plain systolic excursion, 6MWD: six-minute walk distance, ^1^: determined by echocardiography.

**Table 2:** Comparison of clinical data of patients with and without bradycardias in cohort two.

		Patients with Bradycardias	Patients Without Bradycardias	*p*-Value
Patients, *n*		14	208	-
Age, mean (SD)		66 (14)	63 (14)	0.441
Sex, female, *n* (%)		6 (43)	111 (53)	0.446
PH group, *n* (%)	1: IPAH	3 (21)	34 (16)	0.621
	1: HPAH	0 (0)	2 (1)	-
	1: CTD-PAH	1 (7)	12 (6)	0.832
	1: PVOD/PCH-PAH	0 (0)	6 (3)	-
	1: PoH-PAH	0 (0)	1 (0.5)	-
	1: CHD-PAH	0 (0)	9 (4)	-
	2	0 (0)	36 (17)	-
	3	6 (43)	43 (21)	0.527
	4	3 (21)	59 (28)	0.576
	5	1 (7)	6 (3)	0.378
6MWD, m, mean (SD)		332 (112)	328 (132)	0.893
BNP, pg/mL, mean (SD)		133 (114)	285 (337)	0.276
sPAP ^1^, mmHg, mean (SD)		46 (21)	66 (24)	0.024
TAPSE, mm, mean (SD)		19 (3)	18 (5)	0.441
PVR, dyn × sec × cm^−5^, mean (SD)		648 (368)	645 (448)	0.983
Antiarrhythmic drugs, *n* (%)	ß-blocker	2 (14)	53 (26)	0.348
	digitalis	2 (14)	40 (19)	0.755
	verapamil/diltiazem	6 (43)	36 (17)	0.018
	amiodaron	0 (0)	6 (3)	-
	flecainide	0 (0)	1 (0.5)	-
	propafenone	0 (0)	1 (0.5)	-
	ivabradine	0 (0)	1 (0.5)	-
AV block, *n* (%)	I	7 (50)	0 (0)	-
	IIa	1 (7)	0 (0)	-
	IIb	2 (14)	0 (0)	-
	III	1 (7)	0 (0)	-
SA block, *n* (%)	I	0 (0)	0 (0)	-
	II	1 (7)	0 (0)	-
	III	1 (7)	0 (0)	-
	unspecified	1 (7)	0 (0)	-

Legend: AV: atrioventricular, BNP: brain natriuretic peptide, CHD-PAH: PAH associated with congenital heart disease, CTD-PAH: PAH associated with connective tissue disease, HPAH: hereditary PAH, IIa: AV block Mobitz type, IIb: AV block Wenckebach type, IPAH: idiopathic PAH, PAH: pulmonary arterial hypertension, PH: pulmonary hypertension, PoH-PAH: PAH associated with portal hypertension, PVOD/PCH-PAH: PAH with features of venous/capillary involvement, PVR: pulmonary vascular resistance, SA: sinoatrial, SD: standard deviation, sPAP: systolic pulmonary artery pressure, TAPSE: tricuspid annular plain systolic excursion, 6MWD: six-minute walk distance, ^1^: determined by echocardiography.

**Table 3:** Prevalence of bradycardias in patients with pulmonary hypertension.

Author	Cohort 1	Cohort 2	Kanemoto et al. [24]	Bossone et al. [25]	Andersen et al. [26]
Year	2012–2013	2000–2013	1979	2002	2021
Study type	prospective	retrospective	retrospective	prospective	prospective
Number of patients	92	222	101	51	34
PH groups	1, 3, 4, 5	1–5	1	1	1, 4
Prevalence of bradycardia	4%	6.5%	12%	4%	9%
Type of bradycardia	1 AVB II° 3 AVB III°	7 AVB I° 3 AVB II° 1 AVB III° 1 SAB II° 1 SAB III° 1 SAB (unspecified)	6 SB 5 AVB I° 1 AVB II°	2 SB	1 SAB (unspecified) 2 SB
Diagnostic method	72 h Holter-ECG	24 h Holter-ECG	12-lead ECG	12-lead ECG	ICM

Legend: AVB: atrioventricular block, ICM: insertable cardiac monitor, ECG: electrocardiogram, PH: pulmonary hypertension, SAB: sinoatrial block, SB: sinus bradycardia.

## Data Availability

The original contributions presented in the study are included in the article. Further inquiries can be directed to the corresponding author.
